# Epidemiology of influenza in West Africa after the 2009 influenza A(H1N1) pandemic, 2010–2012

**DOI:** 10.1186/s12879-017-2839-1

**Published:** 2017-12-04

**Authors:** Ndahwouh Talla Nzussouo, Jazmin Duque, Adebayo Abel Adedeji, Daouda Coulibaly, Samba Sow, Zekiba Tarnagda, Issaka Maman, Adamou Lagare, Sonia Makaya, Mohamed Brahim Elkory, Herve Kadjo Adje, Paul Alhassan Shilo, Boubou Tamboura, Assana Cisse, Kossi Badziklou, Halima Boubacar Maïnassara, Ahmed Ould Bara, Adama Mamby Keita, Thelma Williams, Ann Moen, Marc-Alain Widdowson, Meredith McMorrow

**Affiliations:** 1 Influenza Division, National Center for Immunization and Respiratory Diseases, Centers for Disease Control and Prevention, Atlanta, GA USA; 2 CTS Global Inc., California, El Segundo USA; 3 Battelle Atlanta, Atlanta, GA USA; 4 National Influenza Reference Laboratory, Federal Ministry of Health, Abuja, Nigeria; 5 Institut National d’Hygiene Publique (INHP), Abidjan, Côte d’Ivoire; 6 Centre National d’Appui à la Lutte Contre la Maladie (CNAM), Centre pour le Développement des Vaccins du Mali (CVD), Bamako, Mali; 7 Institut de Recherche en Sciences de Santé (IRSS), Bobo-Dioulasso, Burkina Faso; 8 Institut National d’Hygiene (INH), Lome, Togo; 9 Centre de Recherche Médicale et Sanitaire (CERMES), Niamey, Niger; 10 Influenza National Reference Laboratory Lakka, Freetown, Sierra Leone; 11 Institut National Recherche en Sante Publique (INRSP), Nouakchott, Mauritanie; 12 Institut Pasteur Cȏte d’Ivoire (IPCI), Abidjan, Côte d’Ivoire; 13 U.S. Public Health Service, Rockville, MD USA; 14 Noguchi Memorial Institute for Medical Research, P.O. Box LG 481, Legon, Accra Ghana

**Keywords:** Epidemiology, Influenza, West Africa

## Abstract

**Background:**

Over the last decade, capacity for influenza surveillance and research in West Africa has strengthened. Data from these surveillance systems showed influenza A(H1N1)pdm09 circulated in West Africa later than in other regions of the continent.

**Methods:**

We contacted 11 West African countries to collect information about their influenza surveillance systems (number of sites, type of surveillance, sampling strategy, populations sampled, case definitions used, number of specimens collected and number of specimens positive for influenza viruses) for the time period January 2010 through December 2012.

**Results:**

Of the 11 countries contacted, 8 responded: Burkina Faso, Cote d’Ivoire, Mali, Mauritania, Niger, Nigeria, Sierra Leone and Togo. Countries used standard World Health Organization (WHO) case definitions for influenza-like illness (ILI) and severe acute respiratory illness (SARI) or slight variations thereof. There were 70 surveillance sites: 26 SARI and 44 ILI. Seven countries conducted SARI surveillance and collected 3114 specimens of which 209 (7%) were positive for influenza viruses. Among influenza-positive SARI patients, 132 (63%) were influenza A [68 influenza A(H1N1)pdm09, 64 influenza A(H3N2)] and 77 (37%) were influenza B. All eight countries conducted ILI surveillance and collected 20,375 specimens, of which 2278 (11%) were positive for influenza viruses. Among influenza-positive ILI patients, 1431 (63%) were influenza A [820 influenza A(H1N1)pdm09, 611 influenza A(H3N2)] and 847 (37%) were influenza B. A majority of SARI and ILI case-patients who tested positive for influenza (72% SARI and 59% ILI) were children aged 0–4 years, as were a majority of those enrolled in surveillance. The seasonality of influenza and the predominant influenza type or subtype varied by country and year.

**Conclusions:**

Influenza A(H1N1)pdm09 continued to circulate in West Africa along with influenza A(H3N2) and influenza B during 2010–2012. Although ILI surveillance systems produced a robust number of samples during the study period, more could be done to strengthen surveillance among hospitalized SARI case-patients. Surveillance systems captured young children but lacked data on adults and the elderly. More data on risk groups for severe influenza in West Africa are needed to help shape influenza prevention and clinical management policies and guidelines.

**Electronic supplementary material:**

The online version of this article (10.1186/s12879-017-2839-1) contains supplementary material, which is available to authorized users.

## Background

Influenza is responsible for substantial morbidity and mortality worldwide. About 3–5 million cases of severe influenza disease and 250,000–500,000 deaths occur every year [1]. Among the most affected risk-groups are young children, pregnant women, the elderly and persons with chronic illnesses. A recent review estimates that each year over 250,000 African children aged less than 5 years are hospitalized with influenza and have influenza-associated hospitalization rates more than 3 times higher than those of children in industrialized countries [2].

Until recently, little was known about influenza epidemiology in in Sub-Saharan Africa [3]. However, since 2006, 30 countries have implemented or improved influenza surveillance systems and 14 countries in Africa have received National Influenza Center (NIC) recognition from the World Health Organization (WHO) [4–7]. Ten of these countries regularly report to the WHO’s Global Influenza Surveillance and Response System (GISRS). These countries have also provided essential information on risk factors for severe influenza-associated illness including HIV and tuberculosis infection, which are relatively common in sub-Saharan Africa [8–11].

Since 2006, in West Africa, Burkina Faso, Cote d’Ivoire, Ghana, Mali, Mauritania, Niger, Nigeria, Sierra Leone, and Togo have established influenza surveillance. The number of surveillance sites and samples collected annually varies considerably by country, but all have shown the ability to establish and maintain sentinel surveillance, or to respond to periodic outbreaks of severe respiratory illness. Furthermore, countries like Cote d’Ivoire and Ghana significantly improved their capacities, allowing their reference laboratories to be recognized by WHO as NICs in 2009 and 2010, respectively. Development of these surveillance systems has provided essential information on the transmission and seasonality of influenza virus circulation in West Africa [12, 13].

In a previous study, we showed that the circulation of the 2009 pandemic influenza strain was delayed in West Africa compared to other sub-regions of the continent [14]; however, data on risk factors for severe disease are limited [15–18]. In this study, we aim to understand and describe the epidemiology of influenza in West Africa during 2010–2012.

## Methods

We contacted Ministries of Health, influenza reference laboratories and/or WHO NICs in 11 countries in West Africa: Burkina Faso, Cameroon, Côte d’Ivoire, Ghana, Mali, Mauritania, Niger, Nigeria, Senegal, Sierra Leone and Togo. We asked for a description of their influenza surveillance systems and any variation from WHO guidelines, and for data on influenza-like-illness (ILI) and severe acute respiratory infection (SARI) from January 1, 2010 through December 31, 2012. Data collected included the number of sentinel surveillance sites (ILI and SARI), the number of specimens tested by week, sex, age group, and the number positive for influenza by type and subtype.

At the time of study, ILI was defined by WHO as an acute respiratory illness with temperature of **≥**38.0 °C and either cough or sore throat, with onset in the last 7 days^1^.s WHO defined SARI among children aged 2 months to <5 years as cough or shortness of breath or difficulty breathing and any sign of severe illness with onset in the last 7 days. For children aged ≥5 years and adults, SARI was defined as a temperature of **≥**38.0 °C and cough with shortness of breath or difficulty breathing with onset in the last 7 days^2^ [19]. In each country, patients presenting to surveillance facilities and who met the case definitions were eligible; it is only after obtaining verbal consent of the patient (or parent/guardian consent for children) that nasopharyngeal and/or oropharyngeal specimens were collected along with clinical and epidemiological data using a case report form. Specimens and corresponding case report forms were then transported from the sentinel sites to the national influenza reference laboratory where samples were tested for influenza viruses by real-time reverse transcription polymerase chain reaction (rRT-PCR), using CDC protocols [20]. In-country data managers collected laboratory and epidemiologic data in central databases.

Data provided for this study were analyzed using the statistical package for social sciences (SPSS) version 20 (IBM Corporation, Armonk, NY) [21]. We defined predominance as >50% of influenza-positive specimens of the same type (and subtype for influenza A viruses) in a given year. We also examined the Spearman correlation between the peak month and the peak percent positive in order to test whether earlier peak corresponds to higher percent positive at the peak.

## Results

### Description of influenza surveillance in western Africa

Of the 11 countries contacted, 8 contributed data for all or a portion of the study period (January 1, 2010 through December 31, 2012): Burkina Faso, Cote d’Ivoire, Mali, Mauritania, Niger, Nigeria, Sierra Leone and Togo. Cote d’Ivoire, Niger and Nigeria had data for the entire study period. Surveillance began in April 2010 for Mali and Togo, in June 2010 for Burkina Faso, in July 2011 for Mauritania and in August 2011 for Sierra Leone. Overall, the eight countries conducted surveillance in 70 sites: 26 SARI and 44 ILI surveillance sites. Burkina Faso conducted ILI surveillance only. Three of the eight countries (Cote d’Ivoire, Mauritania and Sierra Leone) had pediatric sites in addition to sites that enrolled older children and adults. Mali, Mauritania, Sierra Leone and Togo had surveillance sites in their capital cities only and the rest of the countries had surveillance sites both inside and outside of their capital cities (Additional file 1: Table S1).

Countries included in this analysis all used the WHO case definitions for SARI and ILI or slight variations (e.g. lower temperature threshold, different duration of symptoms – see summary in Additional file 2: Supplemental Information). In each country, patients presenting to surveillance facilities who met the case definition were eligible. After verbal consent (or parent/guardian consent for children), nasopharyngeal and/or oropharyngeal specimens were collected along with clinical and epidemiological data using a case report form. In Mali, Nigeria and Togo, both nasopharyngeal and oropharyngeal samples were collected; while only oropharyngeal samples were collected in Burkina Faso and Mauritania; and only nasopharyngeal samples collected in Cote d’Ivoire, Niger and Sierra Leone. For SARI, all consenting, eligible case-patients were enrolled. For ILI cases, the first 4–5 consenting, eligible case-patients were enrolled on weekdays.

Over the period of study, the number of samples collected annually from the 8 participating countries increased from 6289 collected in 2010 to 9112 collected in 2012 (Table 1). Of these, 23,489 samples were from persons from whom age had been collected and further analyses were restricted to this subset. Of the 23,489 samples, Mali collected 8062 (34.3%) samples, Nigeria 6975 (29.7%) samples, Cote d’Ivoire 3405 (14.5%) samples, Niger 1868 (8%) samples, Sierra Leone 1111 (4.7%) samples, Burkina Faso 1009 (4.3%) samples, Togo 963 (4.1%) samples and Mauritania 97 (0.4%) samples (Table 1).

**Table 1:** Influenza test results for influenza-like illness (ILI) and severe acute respiratory illness (SARI) by country, year, and type/subtype in 8 West African countries, 2010–2012

Year	Country	ILI Samples tested	SARI Samples tested	Total ILI positives (%)	Total SARI positives (%)	A(H1N1) (%)	A(H3N2) (%)	B (%)
2010	Burkina Faso	174	0	6 (3.5)	0 (0.0)	3 (1.7)	2 (1.1)	1 (0.6)
	Cote d’Ivoire	898	60	171 (19.0)	1 (1.7)	52 (5.4)	57 (5.9)	63 (6.6)
	Mali	2207	0	70 (3.2)	0 (0.0)	8 (0.4)	44 (2.0)	18 (0.8)
	Mauritania	0	0	0 (0.0)	0 (0.0)	0 (0.0)	0 (0.0)	0 (0.0)
	Niger	298	88	61 (20.5)	8 (9.1)	50 (13.0)	6 (1.6)	13 (3.4)
	Nigeria	1844	633	144 (7.8)	34 (5.4)	104 (4.2)	20 (0.8)	54 (2.2)
	Sierra Leone	0	0	0 (0.0)	0 (0.0)	0 (0.0)	0 (0.0)	0 (0.0)
	Togo	87	0	28 (32.2)	0 (0.0)	21 (24.1)	4 (4.6)	3 (3.4)
	Sub Total 2010	5508	781	480 (8.7)	43 (5.5)	238	133	152
2011	Burkina Faso	453	0	38 (8.4)	0 (0.0)	13 (2.9)	3 (0.7)	22 (4.9)
	Cote d’Ivoire	895	67	553 (61.8)	23 (34.3)	211 (21.9)	58 (6.0)	307 (31.9)
	Mali	2342	0	106 (4.5)	0 (0.0)	26 (1.1)	2 (0.1)	78 (3.3)
	Mauritania	0	0	0 (0.0)	0 (0.0)	0 (0.0)	0 (0.0)	0 (0.0)
	Niger	428	429	35 (8.2)	17 (4.0)	37 (4.3)	11 (1.3)	4 (0.5)
	Nigeria	2075	542	228 (11.0)	28 (5.2)	56 (2.1)	87 (3.3)	113 (4.3)
	Sierra Leone	299	223	39 (13.0)	19 (8.5)	5 (1.0)	53 (10.2)	0 (0.0)
	Togo	335	0	85 (25.4)	0 (0.0)	22 (6.6)	16 (4.8)	47 (14.0)
	Sub Total 2011	6827	1261	1084 (15.9)	87 (7.0)	370	230	571
2012	Burkina Faso	382	0	14 (3.7)	0 (0.0)	5 (1.3)	8 (2.1)	1 (0.3)
	Cote d’Ivoire	1413	72	211 (14.9)	6 (8.3)	113 (7.6)	83 (5.6)	21 (1.4)
	Mali	3513	2	161 (4.6)	0 (0.0)	83 (2.4)	56 (1.6)	22 (0.6)
	Mauritania	60	37	7 (11.7)	0 (0.0)	0 (0.0)	2 (2.1)	5 (5.2)
	Niger	251	373	22 (8.8)	17 (4.6)	0 (0.0)	25 (4.0)	14 (2.2)
	Nigeria	1609	272	149 (9.3)	12 (4.4)	35 (1.9)	70 (3.7)	56 (3.0)
	Sierra Leone	292	297	38 (13.0)	39 (13.1)	23 (3.9)	19 (3.2)	35 (5.9)
	Togo	522	19	115 (22.0)	2 (10.5)	21 (3.9)	49 (9.1)	47 (8.7)
	Sub Total 2012	8042	1070	717 (8.9)	76 (7,1)	280	312	201
	Total	20,375	3114	2281 (11.2)	206 (6.6)	888	675	924

### Characteristics of ILI and SARI cases

Of the 23,489 enrollees, 20,375 ILI and 3114 SARI specimens were collected and processed by the eight participating countries. Among 20,375 ILI case-patients, the majority were children aged 0–4 years (14,775, 72.5%) and 5–14 years (1650, 8.1%). Older children and adults aged 15–64 years (3665, 18.0%) and adults aged 65 years and older (135, 0.7%) represented less than one-fifth of all enrolled participants. Information on age was missing for 150 (0.7%) tested ILI cases. Among 3114 SARI case-patients, 2100 (67.4%) were children aged 0–4 years, 216 (6.9%) children aged 5–14 years, 471 (15.1%) older children and adults aged 15–64 years and 47 (1.5%) adults aged 65 years and older. Age was missing for 280 (9.1%) tested SARI cases. Excluding those with missing age, the distribution of ILI and SARI cases among these age groups was significantly different (*p* < 0.001), with those enrolled in SARI more likely to be children aged 0–4 years and adults aged 65 years and older.

Among the 23,489 samples collected, 9577 (40.8%) were from males, 8807 (37.5%) were from females, and gender was not reported for 5105 (21.7%) case-patients. Of the 20,375 ILI samples, 7977 (39.2%) and 7513 (36.9%) were collected from males and females, respectively. Of the 3114 SARI samples, 1600 (51.4%) and 1294 (41.6%) were collected from males and females respectively. For SARI cases, length of hospitalization and outcome of hospitalization were not reported.

### Influenza prevalence among ILI and SARI cases

Overall 2278 (11.2%) of 20,375 ILI case-patients tested positive for influenza, ranging from 29.2% in Cote d’Ivoire to 4.2% in Mali. Among the countries that also conducted SARI surveillance, 209 (6.7%) of 3114 SARI case-patients tested positive for influenza, ranging from 31.6% in Togo to 4.7% in Niger. A majority of ILI case-patients testing positive for influenza viruses were children aged 0–4 years (1333, 58.5%) and 5–14 years (293, 12.9%). The remaining ILI positive samples were collected from patients aged 15–64 years (611, 26.8%) and patients aged 65 years and older (13, 0.6%). Age was not reported for 28 (1.2%) of influenza-positive ILI case-patients. Among the 209 influenza-positive SARI samples, 151 (72.3%) were collected from children aged 0–4 years, 22 (10.5%) from children aged 5–14 years, 29 (13.9%) from patients aged 15–64 years, 4 (1.9%) from patients aged 65 years and older. Age was not reported for 3 (1.4%) influenza-positive SARI case-patients.

**Fig. 1:**
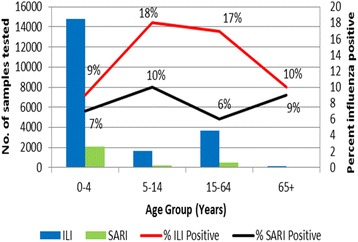
Number of influenza-like illness (ILI) and severe acute respiratory illness (SARI) cases tested and proportion positive for influenza by age group, West Africa, 2010–2012

Although data are limited, the proportion influenza positive among SARI cases did not vary significantly by age group (*p* = 0.29) (Fig. 1); however, children aged 5–14 years and adults aged 15–64 years with ILI had a higher proportion influenza positive than children aged 0–4 years and adults aged 65 years and older (*p* = 0.01).

Of the 2278 ILI positive cases, 1118 (49.1%) and 1131 (49.7%) were collected from males and females, respectively. Of the 209 SARI positive cases, 117 (56.0%) and 88 (42.1%) were collected from males and females respectively. However, gender was not reported for 29 (1.3%) influenza-positive ILI case-patients and four (1.9%) influenza-positive SARI case-patients.

### Influenza types and subtypes and seasonality by country

Among influenza-positive ILI cases, 1431 (62.8%) were influenza A and 847 (37.2%) were influenza B. Among the influenza A positives, 820 (57.3%) were influenza A(H1N1)pdm09 and 611 (42.7%) were influenza A(H3N2). Among influenza-positive SARI cases, 132 (63.2%) were influenza A and 77 (36.8%) were influenza B. Among the influenza A positives, 68 (51.5%) were influenza A(H1N1)pdm09 and 64 (48.5%) were influenza A(H3N2).

**Fig. 2:**
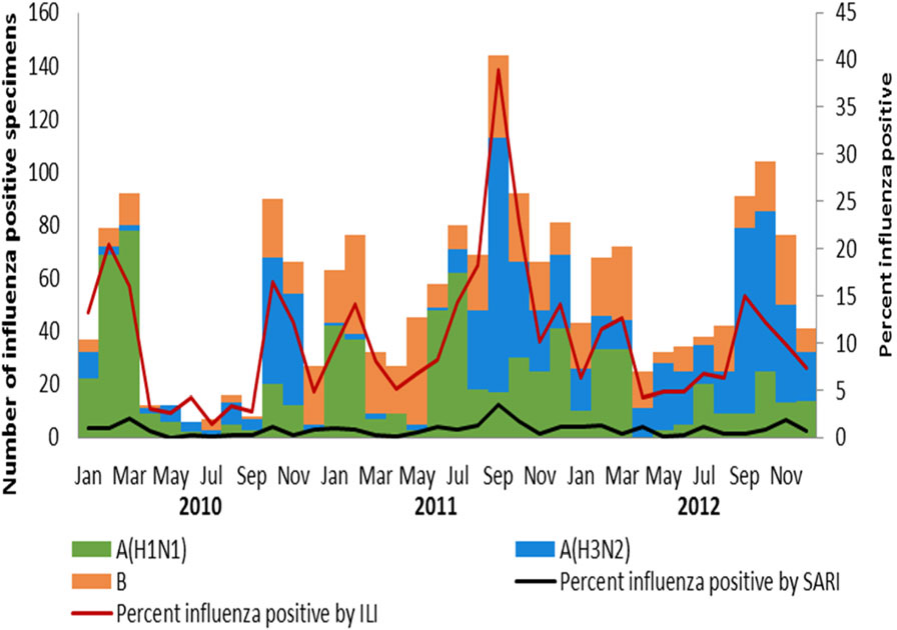
Summary of influenza virus circulation by month in 8 West African countries, 2010–2012

Based on aggregate data from all eight countries, influenza appears to circulate year round in West Africa, with 2 major peaks during the year; the first occurring between January–March (>15% influenza positive) corresponding to the dry/harmattan season and the second peak between August–November (>15% influenza positive) corresponding to the rainy season (Fig. 2, Additional file 3: Figure S1, Additional file 4: Figure S2, Additional file 5: Figure S3, Additional file 6: Figure S4, Additional file 7: Figure S5, Additional file 8: Figure S6, Additional file 9: Figure S7, Additional file 10: Figure S8). There were no significant correlations between the peak month and peak percent influenza positive (*p* = 0.67). The influenza A(H1N1)pdm09 strain began to actively circulate in Cote d’Ivoire, Niger, Togo and Nigeria in early 2010 and was the predominant circulating strain in the latter three; while co-circulation of influenza A(H1N1)pdm09 with influenza A(H3N2) and influenza B was observed in Cote d’Ivoire and Burkina Faso (Table 1). In Mali, influenza A(H3N2) was the predominant circulating subtype in 2010. In 2011, influenza A(H1N1)pdm09 was the predominant circulating subtype only in Niger while influenza A(H3N2) was predominant in Sierra Leone and influenza B viruses were predominant in Mali and Togo. At the same time, all three types/subtypes co-circulated in Burkina Faso, Cote d’Ivoire and Nigeria. In 2012, influenza A(H1N1)pdm09 was no longer the predominant circulating strain in any of the 8 countries. Influenza A(H1N1)pdm09, influenza A(H3N2) and influenza B viruses co-circulated in all countries except in Mauritania and Niger where influenza B and influenza A(H3N2) appeared dominant, respectively.

## Discussion

While useful information has been obtained from influenza surveillance in West Africa, this analysis demonstrates some key weaknesses in influenza surveillance during this time period. There were relatively few samples collected from hospitalized patients with SARI. With few data on hospitalized cases it is difficult to determine risk factors for severe influenza disease, which would help with prioritization of seasonal and pandemic vaccine. Half (4/8) of the countries collected none or less than 50 SARI samples during the 3-year study period. Establishing SARI surveillance has proven to be quite challenging in tertiary hospitals and secondary hospitals as these medical facilities are generally quite understaffed. Medical staffs struggling to provide basic services rarely collect specimens for surveillance purposes. Given budgetary and human resource constraints, having dedicated staff that would focus only or primarily on SARI surveillance and collection of outcome and denominator data may be unrealistic in these facilities without alternative means of support. Integration with other disease surveillance programs is possible, but would require further consideration given the need for laboratory confirmation and stringent handling requirements for virologic specimens.

Additionally, data collected on adults and the elderly are quite limited. During the study period, 80% of ILI and 74% of SARI samples were collected from children aged 0–14 years. Children aged 0–14 years are approximately 43% of the total population of West Africa, given the higher rates of influenza-associated illness seen in children it is not unexpected that they would be a majority of SARI and ILI case-patients [22, 23]; however, without systematic data collection it is difficult to assess these potential biases in enrollment. Collection of data on ages of all respiratory admissions could improve the understanding of potential biases in existing surveillance. In addition, the few number of elderly enrolled through facility-based surveillance suggest the need to identify surveillance sites that primarily serve adults or the use of community-based surveillance in some settings in order to improve representativeness. Collection of data on other risk factors for severe disease and outcomes of hospitalization (e.g. death, discharge) would also be useful to inform policy decisions. Furthermore, data were collected from health facility-based sentinel surveillance. Persons aged 60 and older are less likely to seek care at health facilities in many parts of Africa which may limit the utility of health facility-based surveillance for monitoring severe disease in this age group [24, 25].

The seasonality of influenza and the predominant influenza type or subtype varied by country and year. While most countries detected influenza virus circulation throughout the year, there appear to be two seasonal peaks in influenza virus circulation in January–March and August–November. Data from Eastern Africa, primarily Kenya and Uganda, show that influenza circulates throughout the year; however, seasonal peaks mostly correspond with East African rainy seasons and the Southern Hemisphere winter [26–28] as opposed to the Western African harmattan and rainy seasons. In Southern Africa, influenza seasonality corresponds to the typical Southern Hemisphere winter months of June to August [29]. In addition to defining seasonality, ongoing influenza surveillance in West Africa has also provided reassurance that recent outbreaks of avian influenza A(H5N1) in poultry and wild birds have not resulted in substantial numbers of human cases.

There were several limitations to this study. First, we assessed just 3 years of surveillance. As influenza transmission varies greatly from year to year, 3 years of data are insufficient to understand nuances of transmission dynamics in the countries we included. Also, not all countries conducted surveillance during the entire time period, which limits interpretation of the percentage positive and may account for the broad range of percent positive by country. Likewise, the individual countries have different climates/μ-climates (coastal, tropical, arid, etc.) that may impact influenza virus circulation. Different case definitions and different sampling methods may have further contributed to the wide range of percent influenza positive by country. Selection bias may have also contributed to the wide range of percent influenza positive by country if clinicians chose to enroll case-patients they suspected of having influenza or to exclude those they thought had a different condition instead of enrolling systematically based upon case definitions. Data on population denominators for surveillance would also help estimate the burden of disease and further inform policy decisions around influenza prevention and control.

We found no association between peak month and monthly percent positive. However, because countries provided aggregate monthly data and, not weekly aggregate data, we cannot say if there would be significant correlations using weekly aggregate data. Additionally, for this analysis, we grouped data from ILI and SARI surveillance in all participating countries. Grouping data in this way may have affected the outcomes of this analysis.

## Conclusion

Influenza surveillance rapidly expanded during 2010–2012 in West Africa. Despite the improvements observed, there is a need to strengthen SARI surveillance and to collect more epidemiological data about outcomes of hospitalization, risk factors for influenza-associated hospitalization, and population denominators. These data will help to better understand influenza-associated disease and economic burden in West Africa. Alternative approaches to health facility-based surveillance may be required to identify influenza disease burden in groups with poor access to health facilities. Identifying risk groups and describing the timing of influenza virus circulation will help shape influenza vaccination policies for seasonal and pandemic preparedness.

## Additional files

Additional file 1: Table S1. Summary of influenza surveillance for influenza-like illness (ILI) and severe acute respiratory illness (SARI) by country, West Africa, 2010–2012. (DOCX 12 kb)

Additional file 2: Supplemental Information ILI/SARI Case Definitions and sample strategy for ILI by country. (DOCX 11 kb)

Additional file 3: Figure S1. Aggregate of influenza surveillance for influenza-like illness (ILI) and severe acute respiratory illness (SARI) data in Burkina Faso: 2010–2012. (DOCX 39 kb)

Additional file 4: Figure S2. Aggregate of influenza surveillance for influenza-like illness (ILI) and severe acute respiratory illness (SARI) data in Cote d’Ivoire: 2010–2012. (DOCX 20 kb)

Additional file 5: Figure S3. Aggregate of influenza surveillance for influenza-like illness (ILI) and severe acute respiratory illness (SARI) data in Mali: 2010–2012. (DOCX 18 kb)

Additional file 6: Figure S4. Aggregate of influenza surveillance for influenza-like illness (ILI) and severe acute respiratory illness (SARI) data in Mauritania: 2010–2012. (DOCX 17 kb)

Additional file 7: Figure S5. Aggregate of influenza surveillance for influenza-like illness (ILI) and severe acute respiratory illness (SARI) data in Niger: 2010–2012. (DOCX 18 kb)

Additional file 8: Figure S6. Aggregate of influenza surveillance for influenza-like illness (ILI) and severe acute respiratory illness (SARI) data in Nigeria: 2010–2012. (DOCX 18 kb)

Additional file 9: Figure S7. Aggregate of influenza surveillance for influenza-like illness (ILI) and severe acute respiratory illness (SARI) data in Sierra Leone: 2010–2012. (DOCX 37 kb)

Additional file 10: Figure S8. Aggregate of influenza surveillance for influenza-like illness (ILI) and severe acute respiratory illness (SARI) data in Togo: 2010–2012. (DOCX 40 kb)

## Abbreviations

CDC: Centers for Disease Control and Prevention
GISRS: Global Influenza Surveillance and Response System
H: Hemagglutinin
HIV: Human immunodeficiency virus
IBM: International Business Machines Corporation
ILI: Influenza-like illness
N: Neuraminidase
NIC: National Influenza Center
NY: New York
pdm: Pandemic
rRT-PCR: Real-time reverse transcription polymerase chain reaction
SARI: Severe acute respiratory illness
SPSS: Statistical package for social sciences
WHO: World Health Organization

## Footnotes

1 Since 2013, the WHO ILI case definition was amended to an acute respiratory infection with measured fever of ≥38 C° and cough with onset within the last 10 days.

2 The new WHO SARI case definition is an acute respiratory infection with history of fever or measured fever of ≥38 C° and cough with onset within the last 10 days requiring hospitalization.
