# Supplementary material for: Epidemiology of influenza in West Africa after the 2009 influenza A(H1N1) pandemic, 2010–2012
Source: BMC Infect Dis. 2017 Dec 4;17:745. doi: 10.1186/s12879-017-2839-1 (PMC5716025; doi:10.1186/s12879-017-2839-1)
Supplement: Supplementary file 1 — Summary of influenza surveillance for influenza-like illness (ILI) and severe acute respiratory illness (SARI) by country, West Africa, 2010–2012. (DOCX 12 kb) [file 12879_2017_2839_MOESM1_ESM.docx]

**Supplemental Table:**

Table 1 Summary of influenza surveillance for influenza-like illness (ILI) and severe acute respiratory illness (SARI) by country, West Africa, 2010-2012

| **Country** | **Date Surveillance Started** | **Number of Sentinel Sites** | | | **Pediatric only (2mo-15yrs** | **Number of Specimens Tested** | | **Number (%) of Influenza-Positive Specimens** | |
| --- | --- | --- | --- | --- | --- | --- | --- | --- | --- |
|  |  | Located in the capital city | ILI | SARI |  | ILI | SARI | ILI | SARI |
| **Burkina Faso** | June 2010 | 2 | 6 | 0 | 0 | 1009 | 0 | 58 (5.7) | 0 (0.0) |
| **Cote d’Ivoire** | January 2008 | 11 | 11 | 8* | 1 | 3206 | 199 | 935 (29.2) | 30 (15.1) |
| **Mali** | April 2010 | 8 | 7 | 1 | 0 | 8060 | 2 | 337 (4.2) | 0 (0.0) |
| **Mauritania** | December 2011 | 2 | 1 | 1 | 1 | 60 | 37 | 7 (11.7) | 0 (0.0) |
| **Niger** | April 2009 | 4 | 7* | 7 | 0 | 977 | 890 | 118 (12.1) | 42 (4.7) |
| **Nigeria** | April 2008 | 1 | 4* | 4 | 0 | 5528 | 1447 | 521 (9.4) | 74 (5.1) |
| **Sierra Leone** | August 2011 | 4 | 4* | 4 | 1 | 591 | 520 | 77 (13.0) | 58 (11.2) |
| **Togo** | April 2010 | 4 | 4 | 1* | 0 | 944 | 19 | 225 (23.8) | 5 (26.3) |

* ILI and SARI sites located within the same health facility
