# Supplementary material for: Epidemiology of influenza in West Africa after the 2009 influenza A(H1N1) pandemic, 2010–2012
Source: BMC Infect Dis. 2017 Dec 4;17:745. doi: 10.1186/s12879-017-2839-1 (PMC5716025; doi:10.1186/s12879-017-2839-1)
Supplement: Supplementary file 2 — Supplemental Information ILI/SARI Case Definitions and sample strategy for ILI by country. (DOCX 11 kb) [file 12879_2017_2839_MOESM2_ESM.docx]

**Supplemental Information:**

**ILI/SARI Case Definitions and sample strategy for ILI by country**

In **Burkina Faso**, the same ILI/SARI WHO case definitions was used throughout the study period. Patients were enrolled when they met the case definition and presented at the sentinel site within seven days following symptoms’ onset.

In **Cote d’Ivoire**, the ILI/SARI case definitions considered fever > 38.0 °C and considered a time frame of seven days after symptoms’ onset. These case definitions did not change during the study period (2010 – 2012).

In **Mali**, the surveillance focused primarily only on young children aged from 0-6 months and on pregnant women for both ILI/SARI during the study period, and considered history of fever. Also, presentation at the sentinel site within seven days after of symptoms’ onset was considered for enrollment.

In **Niger**, the case definitions considered fever ≥37.5°C for ILI and SARI (patient ≥ 5 years) and presentation at the sentinel sites within seven days after symptoms’ onset was also considered for enrollment. These case definitions didn’t change throughout the study period.

In **Nigeria**, the ILI case definition considered measured fever ≥38.0°C while a temperature > 38.0°C was considered for SARI case definition (patient ≥ 5 years). Patients were enrolled only when they presented at the sentinel site within fourteen days following symptoms’ onset. Additionally, the following inclusion criteria were considered in Nigeria;

• 4 ILI cases on each of the first 4 workdays of each week had their epidemiological data and respiratory specimens collected.

• All ILI seen in the outpatient department on the first 4 workdays of each week were counted.

• All SARI (in-patients) were counted, epidemiological data and specimen collected

In **Togo**, the WHO case definition for ILI was used except that only a three-day time frame after symptoms’ onset was considered until May 2012 when it was then changed to seven days. The case definition was slightly modified in May 2012 to include history of fever. The SARI case definition for patient ≥ 5 years in Togo considered both measured fever and history of fever throughout the study period.
