# Supplementary figures and images for: Epidemiology of influenza in West Africa after the 2009 influenza A(H1N1) pandemic, 2010–2012
Source: BMC Infect Dis. 2017 Dec 4;17:745. doi: 10.1186/s12879-017-2839-1 (PMC5716025; doi:10.1186/s12879-017-2839-1)

**
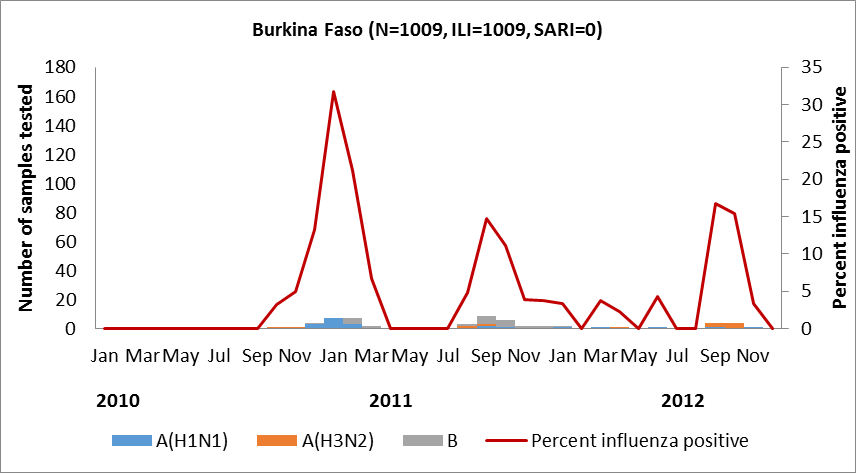
**

Supplement: Supplementary file 3 — Aggregate of influenza surveillance for influenza-like illness (ILI) and severe acute respiratory illness (SARI) data in Burkina Faso: 2010–2012. (DOCX 39 kb) [file 12879_2017_2839_MOESM3_ESM.docx]

**
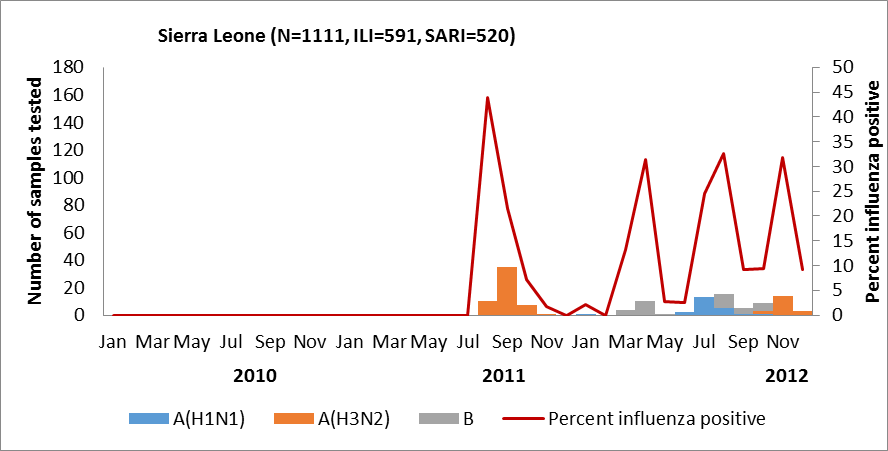
**

Supplement: Supplementary file 9 — Aggregate of influenza surveillance for influenza-like illness (ILI) and severe acute respiratory illness (SARI) data in Sierra Leone: 2010–2012. (DOCX 37 kb) [file 12879_2017_2839_MOESM9_ESM.docx]

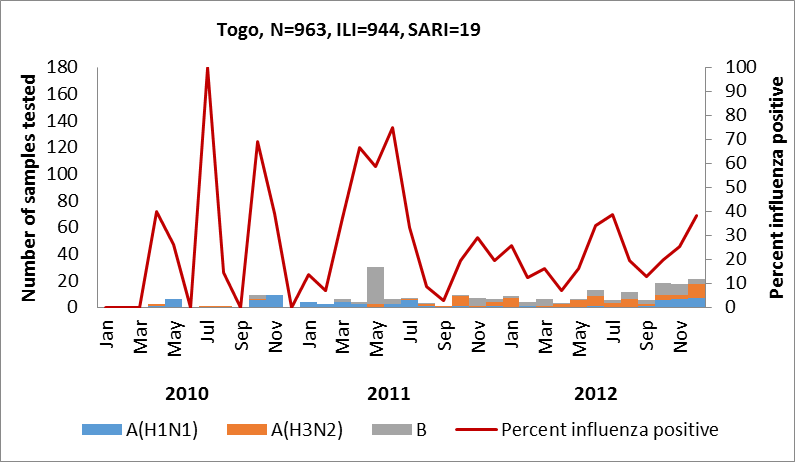

Supplement: Supplementary file 10 — Aggregate of influenza surveillance for influenza-like illness (ILI) and severe acute respiratory illness (SARI) data in Togo: 2010–2012. (DOCX 40 kb) [file 12879_2017_2839_MOESM10_ESM.docx]
