# Supplementary material for: Epidemiology of influenza in West Africa after the 2009 influenza A(H1N1) pandemic, 2010–2012
Source: BMC Infect Dis. 2017 Dec 4;17:745. doi: 10.1186/s12879-017-2839-1 (PMC5716025; doi:10.1186/s12879-017-2839-1)
Supplement: Supplementary file 5 [file 12879_2017_2839_MOESM5_ESM.docx]

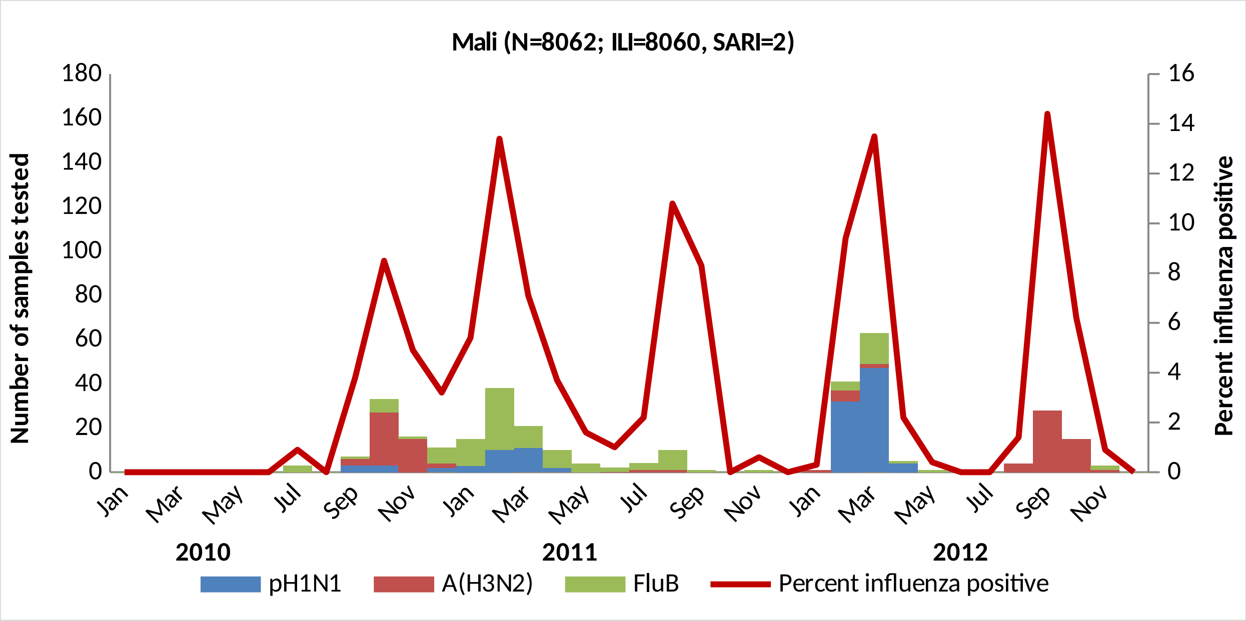

Mali (N=8062; ILI=8060, SARI=2)
Number of samples tested
Percent influenza positive
180
160
140
120
100
80
60
40
20
0
16
14
12
10
8
6
4
2
0
Jan
Mar
May
Jul
Sep
Nov
2010
2011
2012
pH1N1
A(H3N2)
FluB
Percent influenza positive
